# Epidemiology, Ventilatory Patterns, and Outcomes in Acute Hypoxemic Respiratory Failure Among ICU Patients Requiring Respiratory Support: A Registry-Based Cohort Study

**DOI:** 10.1097/CCM.0000000000007284

**Published:** 2026-08-12

**Authors:** Stephan von Düring, Ewan Goligher, Andre Carlos Kajdacsy-Balla Amaral, Gordon D. Rubenfeld, Niall D. Ferguson, Martin Urner, Eddy Fan

**Affiliations:** 1 Division of Intensive Care Medicine, Department of Acute Care Medicine, Geneva University Hospitals, Geneva, Switzerland.; 2 Department of Anaesthesiology, Pharmacology, Intensive Care and Emergency Medicine, Faculty of Medicine, University of Geneva, Geneva, Switzerland.; 3 Interdepartmental Division of Critical Care Medicine, University of Toronto, Toronto, ON, Canada.; 4 Department of Physiology, University of Toronto, Toronto, ON, Canada.; 5 Toronto General Hospital Research Institute, Toronto, ON, Canada.; 6 Department of Critical Care Medicine, Sunnybrook Health Sciences Centre, Toronto, ON, Canada.; 7 Institute of Health Policy, Management and Evaluation (IHPME), University of Toronto, Toronto, ON, Canada.; 8 Department of Medicine, Division of Respirology and Critical Care, University Health Network, Toronto, ON, Canada.; 9 Department of Anesthesiology and Pain Medicine, University of Toronto, Toronto, ON, Canada.

**Keywords:** artificial respiration, hypoxemia, intensive care units, respiratory distress syndrome, respiratory insufficiency

## Abstract

**OBJECTIVES:**

Acute hypoxemic respiratory failure (AHRF) is a leading cause of ICU admission and is associated with significant morbidity and mortality. Yet, epidemiological data on AHRF remain limited, and current management strategies largely derive from studies on acute respiratory distress syndrome (ARDS). We examined the prevalence of AHRF among ICU patients receiving supplemental oxygen or respiratory support for at least 4 hours, as well as initial respiratory support strategies and associated outcomes.

**DESIGN:**

Multicenter registry-based cohort study, 2014–2023.

**SETTING:**

Nine university of Toronto affiliated ICUs.

**Patients:**

Adult ICU patients receiving supplemental oxygen or respiratory support who met AHRF criteria, defined as Pao_2_/Fio_2_ less than or equal to 300 mm Hg or, if Pao_2_ was not available, peripheral oxygen saturation (SpO_2_)/Fio_2_ less than or equal to 315, within the first 24 hours of ICU admission.

**INTERVENTIONS:**

None.

**MEASUREMENTS AND MAIN RESULTS:**

The primary outcome was AHRF prevalence. Secondary outcomes included ICU length of stay, invasive mechanical ventilation (IMV) duration, ventilator-free days, and ICU mortality, stratified by AHRF severity. Of 21,714 patients recorded in the registry, 10,832 (50%) met AHRF criteria, of whom 76% required IMV. Most received lung-protective ventilation. With increasing AHRF severity, ICU length of stay and IMV duration increased, while ventilator-free days and probability of ICU discharge at 30 days decreased. ICU mortality was 23% overall and 47% among patients on IMV with severe AHRF.

**CONCLUSIONS:**

AHRF is common early after ICU admission among patients requiring supplemental oxygen or respiratory support and is frequently managed using ARDS-based strategies. A pragmatic, standardized, and operationally feasible definition of AHRF may improve patient recognition, enable comparisons across studies, and guide future research.

**KEY POINTS**

**Question**: What is the prevalence of acute hypoxemic respiratory failure (AHRF) within the first 24 hours of ICU admission among patients receiving supplemental oxygen or respiratory support, and what are the associated respiratory support strategies and outcomes?

**Findings**: In this multicenter registry-based cohort study, 50% of ICU patients developed AHRF within the first 24 hours, 76% required invasive mechanical ventilation and were frequently managed using acute respiratory distress syndrome-based strategies, and mortality increased with severity, reaching 47% in severe cases.

**Meaning**: AHRF is highly prevalent among ICU patients requiring supplemental oxygen or respiratory support and requires a standardized, pragmatic definition to improve recognition, enable study comparisons, and guide future research.

Patients with acute hypoxemic respiratory failure (AHRF) frequently require respiratory support, and experience significant morbidity and mortality (1, 2). AHRF is often defined by new-onset oxygenation failure with a Pao_2_/Fio_2_ less than or equal to 300 mm Hg (3). It affects 15–18% of ICU admissions and up to 35% of patients receiving invasive mechanical ventilation (IMV) (1, 2, 4, 5). The estimated ICU prevalence of AHRF is around 50%, with most patients presenting with mild hypoxemia (Pao_2_/Fio_2_ 201–300 mm Hg) (3, 6). Data suggest that up to 40% of AHRF patients are managed with high-flow nasal cannula (HFNC) or noninvasive ventilation (NIV), even in severe hypoxemia (Pao_2_/Fio_2_ ≤ 100 mm Hg) (3). ICU mortality averages 27%, and increases with AHRF severity, while in-hospital mortality averages 35% (2, 3, 7).

Management strategies for AHRF are largely derived from acute respiratory distress syndrome (ARDS) research, although findings may not be generalizable to the broader AHRF population (8, 9). ARDS remains under-recognized in up to 40% of eligible patients (10), mainly due to: 1) interobserver variability in identifying bilateral opacities on chest imaging (11); 2) reliance on Pao_2_ from arterial blood gas (ABG); and 3) the PEEP threshold in the Berlin definition excludes otherwise similar patients receiving HFNC or standard oxygen therapy, although recent global and European Society of Intensive Care Medicine (ESICM) guidelines recognize nonintubated ARDS on HFNC and allow for peripheral oxygen saturation to Fio_2_ (Spo_2_/Fio_2_) ratio when ABG is unavailable (8, 9).

In contrast, AHRF is more inclusive and can be identified independently of oxygen delivery mode or ABG availability. Although several multicenter cohorts have described acute respiratory failure using Pao_2_/Fio_2_-based definitions (2, 3, 6, 12), few have operationalized AHRF using the Spo_2_/Fio_2_ ratio across contemporary respiratory support strategies. The Spo_2_/Fio_2_ ratio has been proposed as a reliable, noninvasive alternative; however, clinical data supporting its use remain limited (13, 14). As a result, AHRF epidemiology remains heterogeneous due to the absence of a consensus definition, and few cohorts have characterized this broader population across contemporary modes of respiratory support (2, 5, 15).

In this large registry-based cohort across nine ICUs, we quantify early AHRF prevalence and describe respiratory support strategies and outcomes. We applied a pragmatic oxygenation-based definition within 24 hours of ICU admission, using Pao_2_/Fio_2_ or Spo_2_/Fio_2_ independent of the mode of respiratory support. This approach reflects contemporary practice across both nonintubated and intubated patients. Leveraging a detailed multicenter database spanning academic and community ICUs, we deliver a large-scale characterization of AHRF in routine ICU practice to inform clinical care and guide future research.

## MATERIALS AND METHODS

### Data Source and Study Population

This multicenter, registry-based cohort study used data from the Toronto Intensive Care Observational Registry (iCORE), which included all adult patients (≥ 18 yr) admitted between April 2014 and January 2023 to one of nine University of Toronto-affiliated ICUs who received supplemental oxygen via low-flow nasal cannula or any form of respiratory support, classified as either noninvasive (HFNC, continuous positive airway pressure [CPAP], or NIV) or IMV, for at least 4 hours. These ICUs are part of academic and community hospitals across the Greater Toronto Area. Data were prospectively collected once daily at 8 am and included clinical variables, ventilation parameters, blood gas, and laboratory measurements. IMV was defined as positive pressure ventilation delivered via endotracheal tube (oral, nasal) or tracheostomy, and NIV as positive pressure ventilation delivered via face mask, nasal mask, or helmet.

We included all patients with AHRF, defined as Pao_2_/Fio_2_ less than or equal to 300 mm Hg or, if Pao_2_ was not available, an Spo_2_/Fio_2_ less than or equal to 315, within the first 24 hours of ICU admission (3, 4). Patients with a Pao_2_/Fio_2_ greater than 300 were excluded regardless of Spo_2_/Fio_2_ values. For patients with multiple ICU admissions during the same hospital stay, only data from the initial admission were included. This project was approved by the Research Ethics Board from the University Health Network (Protocol N°. 12-5747.24, Study title “Toronto Intensive Care Registry [iCORE] Project,” approval date: April 3, 2014), and was conducted in accordance with the ethical standards of the institutional committee and the Declaration of Helsinki. Informed consent was waived given the retrospective design and data were de-identified.

### Severity of AHRF and Clinical Measurements

AHRF severity was categorized using the same Pao_2_/Fio_2_ thresholds as the Berlin definition for ARDS: mild (201–300 mm Hg), moderate (101–200 mm Hg), and severe (≤ 100 mm Hg) (16). When Pao_2_/Fio_2_ ratios were unavailable, severity was assigned using Spo_2_/Fio_2_ thresholds: mild (236–315), moderate (149–235), and severe (≤ 148) (17). This hierarchical classification reflects clinical practice, where Pao_2_-based categorization is prioritized when available. Fio_2_ was recorded directly from the device for HFNC, CPAP, NIV, and IMV. For low-flow supplemental oxygen via nasal cannula, Fio_2_ was estimated using conventional bedside conversion: 1 L/min equals 0.24, +0.04 per additional L/min (18). For patients receiving IMV, we described ventilation parameters, static respiratory system elastance (E_RS_), and use of adjunctive therapies within the first 30 days of their ICU stay, stratified by AHRF severity and E_RS_ normalized to predicted body weight (PBW), with low normalized E_RS_ defined as less than 2.5 and high as greater than or equal to 2.5 cm H_2_O/(mL/kg PBW) (19). When recorded, driving pressure (∆P) was calculated as airway plateau pressure (P_plat_) minus total PEEP. Mechanical power was calculated as respiratory rate × tidal volume (Vt) × (peak inspiratory airway pressure–[∆P]/2) × 0.098) (20). Ventilatory ratio, a surrogate of physiologic dead space fraction, was calculated as (minute ventilation × Paco_2_)/(PBW × 100 × 37.5) (21).

### Outcomes

The primary outcome was AHRF prevalence within 24 hours of ICU admission. This window was selected because iCORE data are recorded once daily at a standardized time point, allowing consistent ascertainment of early hypoxemia at ICU presentation. Secondary outcomes were focused on patients receiving IMV and included ventilation parameters, interventions and adjunctive therapies up to day 30, ICU length of stay (LOS), duration of IMV, ICU mortality, and ventilator-free days (VFDs). Patients were followed from the time they met AHRF criteria within 24 hours of admission until ICU discharge or death in the ICU, whichever occurred first. Similar to previous work, VFDs through day 28 were defined for patients intubated for more than 48 hours and calculated as 28 minus the number of calendar days intubated (22, 23). For patients intubated longer than 28 days, VFD was set to 0; for patients who died within 90 days after IMV initiation, VFD was set to –1 (22).

### Statistical Analysis

Descriptive statistics were used to characterize the patient population. Continuous variables were summarized as median and interquartile range (IQR), and categorical variables as counts or percentages (%). The 30-day cumulative probability of death was estimated using a competing risk regression, where ICU discharge was considered a competing event precluding patients from dying in the ICU.

Given the descriptive nature of the study, we conducted a complete case analysis without making any assumptions about missing data. A two-sided *p* value of less than 0.05 was considered statistically significant. Analyses were performed using R, Version 4.5.0 (R Foundation for Statistical Computing, Vienna, Austria) and reported according to the Strengthening the Reporting of Observational Studies in Epidemiology guidelines (https://links.lww.com/CCM/I35).

### Sensitivity and Subgroup Analyses

To examine whether our results were consistent under more restrictive case definitions and explore potential sources of misclassification, we performed two sensitivity analyses: 1) restricted to patients with Spo_2_ less than 97%, as values on the flat portion of the oxyhemoglobin dissociation curve correspond to a wide range of Pao_2_ levels and cannot reliably identify true hypoxemia and 2) restricted to patients on IMV with conditions more likely to be associated with ARDS, including pneumonia, sepsis, trauma, aspiration, postoperative respiratory failure, and acute respiratory failure not attributable to COPD. As a subgroup analysis, we examined patients on IMV with Pao_2_/Fio_2_ less than or equal to 150 mm Hg to assess the incidence of prone positioning within the first 30 days, aiming to explore adherence to ARDS guideline-recommended practices in patients with moderate-to-severe hypoxemia.

## RESULTS

### Prevalence of AHRF and Baseline Characteristics

Among the 21,714 critically ill patients receiving supplemental oxygen or respiratory support, 10,832 (50%) met criteria for AHRF and were included in this study (**Fig. 1**). Of these, 3831 (35%) had Spo_2_/Fio_2_ measurements only, and 34 (< 1%) had recordings of Pao_2_/Fio_2_ only. Among patients with paired measurements, severity classification showed good agreement (**eTable 1**, https://links.lww.com/CCM/I35). Median (IQR) age was 63 years (51–73 yr), and 37% were women (**Table 1**). The most common comorbidities were cardiovascular disease, respiratory disease, and diabetes. The primary indication for initiating respiratory support were acute respiratory failure from all causes and altered level of consciousness. Patients with a more severe AHRF had higher Acute Physiology and Chronic Health Evaluation III and Sequential Organ Failure Assessment scores.

**TABLE 1.** Baseline Characteristics of Patients With Acute Hypoxemic Respiratory Failure, Stratified by Severity

Characteristic	Overall (*n* = 10,832)	Stratification by Acute Hypoxemic Respiratory Failure Severity^a^		
		Mild (*n* = 5,466)	Moderate (*n* = 4,070)	Severe (*n* = 1,296)
Baseline characteristics				
Age, yr	63 (51–73)	63 (52–73)	63 (51–73)	61 (50–72)
Female sex, *n*	4,019 (37%)	1,998 (37%)	1,520 (37%)	501 (39%)
Body mass index, kg/m^2^	27 (23–32)	27 (23–32)	28 (23–33)	27 (23–33)
Acute Physiology and Chronic Health Evaluation III score	75 (56–93)	70 (53–89)	78 (60–96)	83 (62–108)
Sequential Organ Failure Assessment score	5 (3–8)	5 (2–7)	6 (3–9)	7 (4–10)
Baseline comorbidities				
Cardiovascular disease, *n*	4,016 (37%)	2,125 (39%)	1,456 (36%)	435 (34%)
Respiratory disease, *n*	2,761 (25%)	1,324 (24%)	1,134 (28%)	303 (23%)
Diabetes, *n*	2,626 (24%)	1,338 (24%)	972 (24%)	316 (24%)
Active neoplasm, *n*	1,102 (10%)	485 (8.9%)	429 (11%)	188 (15%)
Chronic kidney disease, *n*	962 (8.9%)	476 (8.7%)	366 (9.0%)	120 (9.3%)
Liver failure, *n*	729 (6.7%)	388 (7.1%)	257 (6.3%)	84 (6.5%)
Immunosuppression, *n*	579 (5.3%)	236 (4.3%)	222 (5.5%)	121 (9.3%)
Indication for respiratory support				
Acute respiratory failure, *n*	7,808 (72%)	3,803 (70%)	3,010 (74%)	995 (77%)
Altered level of consciousness, *n*	3,074 (28%)	1,715 (31%)	1,065 (26%)	294 (23%)
Acute exacerbation of chronic pulmonary disease, *n*	374 (3.5%)	162 (3.0%)	178 (4.4%)	34 (2.6%)
Neuromuscular causes, *n*	57 (0.5%)	35 (0.6%)	15 (0.4%)	7 (0.5%)
Respiratory conditions				
Pao_2_/Fio_2_, mm Hg	196 (140–248)	250 (225–275)	156 (130–180)	81 (67–91)
Peripheral oxygen saturation/Fio_2_	225 (163–250)	247 (240–282)	192 (160–213)	100 (94–122)
Noninvasive respiratory support, *n*	1,054 (9.7%)	381 (7.0%)	442 (11%)	231 (18%)
High-flow nasal cannula, *n*	310 (2.9%)	49 (0.9%)	138 (3.4%)	123 (9.5%)
Continuous positive airway pressure, *n*	343 (3.2%)	194 (3.5%)	118 (2.9%)	31 (2.4%)
Noninvasive ventilation, *n*	401 (3.7%)	138 (2.5%)	186 (4.6%)	77 (5.9%)
Invasive mechanical ventilation, *n*	8,280 (76%)	4,065 (74%)	3,247 (80%)	968 (75%)

a Three thousand eight hundred thirty-one patients (35%) had missing Pao_2_/Fio_2_ at baseline and were classified using peripheral oxygen saturation (Spo_2_)/Fio_2_.

Values are reported as medians (interquartile range) or counts (%). Baseline data were collected at 8 am within the first 24 hr of ICU admission. Acute hypoxemic respiratory failure severity was defined using Pao_2_/Fio_2_ thresholds. When unavailable, Spo_2_/Fio_2_ thresholds were used.

**Figure 1.**
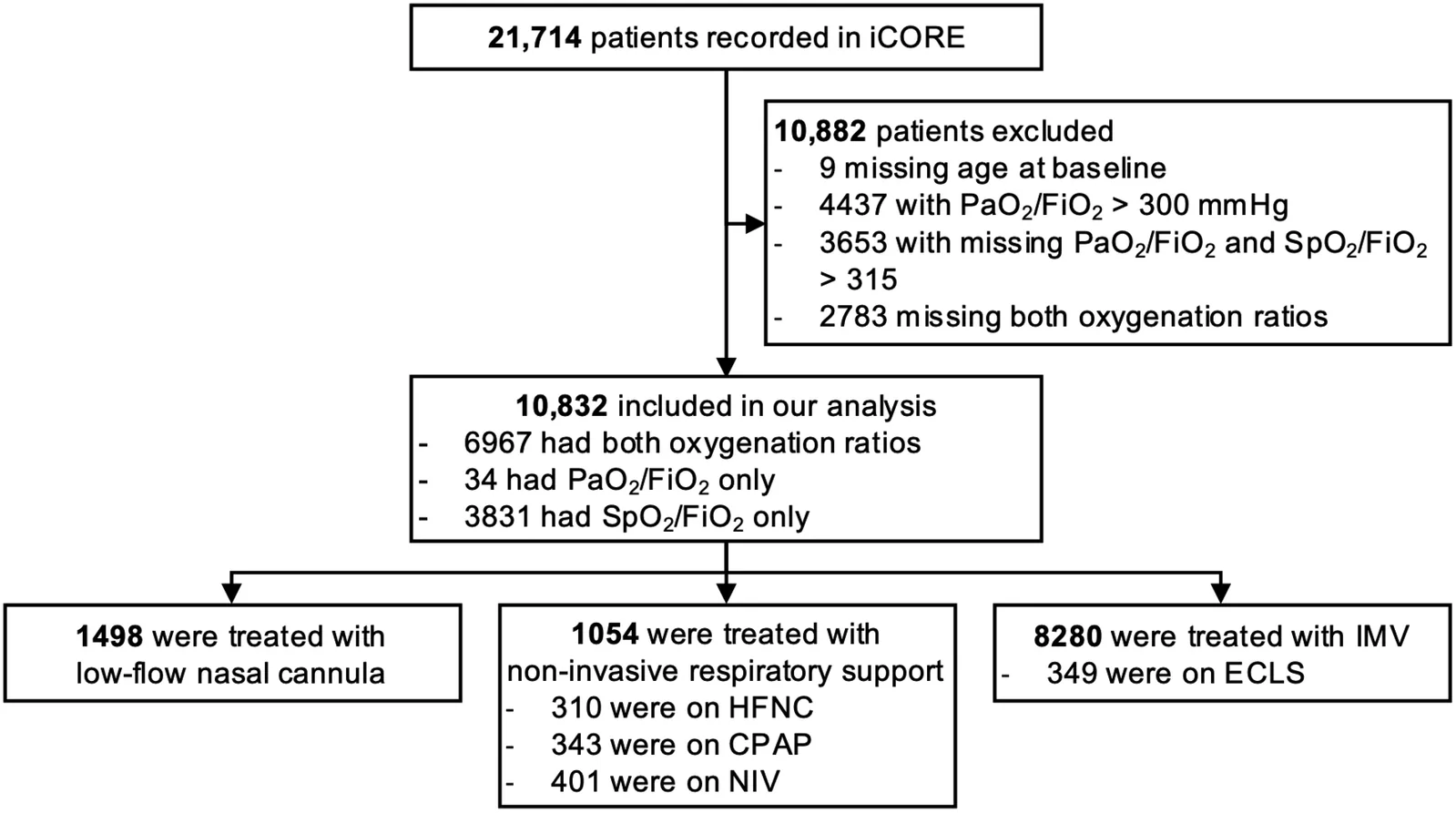
Study flowchart. Among the 21,714 patients initially recorded in our registry, 10,832 patients met our criteria for acute hypoxemic respiratory failure (AHRF) and subsequently were included in our analysis. CPAP = continuous positive airway pressure, ECLS = extracorporeal life support, HFNC = high-flow nasal cannula, iCORE = Toronto Intensive Care Observational Registry, IMV = invasive mechanical ventilation, NIV = noninvasive ventilation, Spo_2_ = peripheral oxygen saturation.

### Ventilation Parameters, Interventions, and Adjunctive Therapies in Patients on IMV

A total of 8280 (76%) patients received IMV within 24 hours, of which 659 (8%) had ARDS documented in the registry (**eTable 2**, https://links.lww.com/CCM/I35). Among patients with available measurements, most were ventilated within lung-protective limits recommended for ARDS management, with a median (IQR) Vt of 6.5 mL/kg PBW (5.9–7.8 mL/kg PBW; **Table 2**; **Fig. 2**; **eFig. 1**, https://links.lww.com/CCM/I35). Pressure-control was the most frequently used mode (37%), especially in more severe AHRF. Among patients receiving volume-controlled ventilation, P_plat_ and ∆P were recorded in 38%, of whom 97% had a P_plat_ less than or equal to 30 cm H_2_O and 78% had a ∆p less than or equal to 14 cm H_2_O. ∆P increased with severity, from a median (IQR) of 10 cm H_2_O (8–13 cm H_2_O) with E_RS_ less than 2.5–17 (15–19) with E_RS_ greater than or equal to 2.5 cm H_2_O/(mL/kg PBW).

**TABLE 2.** Ventilatory Characteristics of Patients on Invasive Mechanical Ventilation With Acute Hypoxemic Respiratory Failure, Stratified by Severity

Characteristic	Overall (*n* = 8280)	Stratification by Acute Hypoxemic Respiratory Failure Severity^a^		
		Mild (*n* = 4065)	Moderate (*n* = 3247)	Severe (*n* = 968)
Blood gases				
Arterial pH	7.35 (7.28–7.40)	7.37 (7.31–7.41)	7.34 (7.27–7.40)	7.28 (7.18–7.36)
Paco_2_, mm Hg	41 (35–47)	39 (35–45)	42 (36–49)	47 (40–56)
Spo_2_ or arterial oxygen saturation, %	97 (94–98)	97 (96–99)	96 (94–98)	94 (90–96)
Pao_2_/Fio_2_, mm Hg	196 (140–247)	250 (225–275)	155 (130–180)	81 (67–90)
Spo_2_/Fio_2_	200 (161–245)	245 (237–277)	190 (158–200)	100 (93–122)
Ventilator parameters				
Fio_2_, %	50 (40–60)	40 (35–40)	50 (50–60)	95 (80–100)
Set respiratory rate, breaths/min	18 (0–24)	16 (0–22)	20 (0–26)	24 (18–30)
Tidal volume, mL/kg PBW	6.5 (5.9–7.8)	6.6 (5.9–7.9)	6.5 (5.8–7.7)	6.3 (5.6–7.4)
Set positive end-expiratory pressure, cm H_2_O	8 (5–10)	5 (5–8)	8 (6–10)	10 (8–12)
Peak inspiratory pressure, cm H_2_O	22 (17–28)	20 (15–24)	24 (19–30)	29 (24–34)
Plateau pressure, cm H_2_O	19 (15–23)	18 (15–21)	20 (16–24)	21 (13–26)
Driving pressure, cm H_2_O	11 (8–14)	11 (8–13)	11 (8–14)	11 (5–16)
Mechanical power, J/min	20 (15–28)	17 (13–23)	22 (16–30)	26 (17–36)
Ventilatory ratio	1.59 (1.25–2.04)	1.48 (1.17–1.83)	1.67 (1.32–2.13)	2.09 (1.61–2.75)
Elastance of the respiratory system, cm H_2_O/(mL/kg PBW)	1.72 (1.28–2.24)	1.67 (1.30–2.10)	1.79 (1.31–2.30)	1.75 (0.94–2.35)
Pressure-controlled ventilation, *n*	3040 (37%)	1312 (32%)	1267 (39%)	461 (48%)
Volume-controlled ventilation, *n*	2506 (30%)	1109 (27%)	1030 (32%)	367 (38%)
Pressure-support ventilation, *n*	2633 (32%)	1610 (40%)	911 (28%)	112 (12%)
Other ventilation modes, *n*	71 (0.9%)	28 (0.7%)	30 (0.9%)	13 (1.3%)

PBW = predicted body weight, Spo_2_ = peripheral oxygen saturation.

a Two thousand four hundred fifty-five patients (30%) had missing Pao_2_/Fio_2_ at baseline and were classified using Spo_2_/Fio_2_.

Values are reported as medians (interquartile range) or counts (%). Baseline data were collected at 8 am within the first 24 hr of ICU admission. Acute hypoxemic respiratory failure severity was defined using Pao_2_/Fio_2_ thresholds. When unavailable, Spo_2_/Fio_2_ thresholds were used.

**Figure 2.**
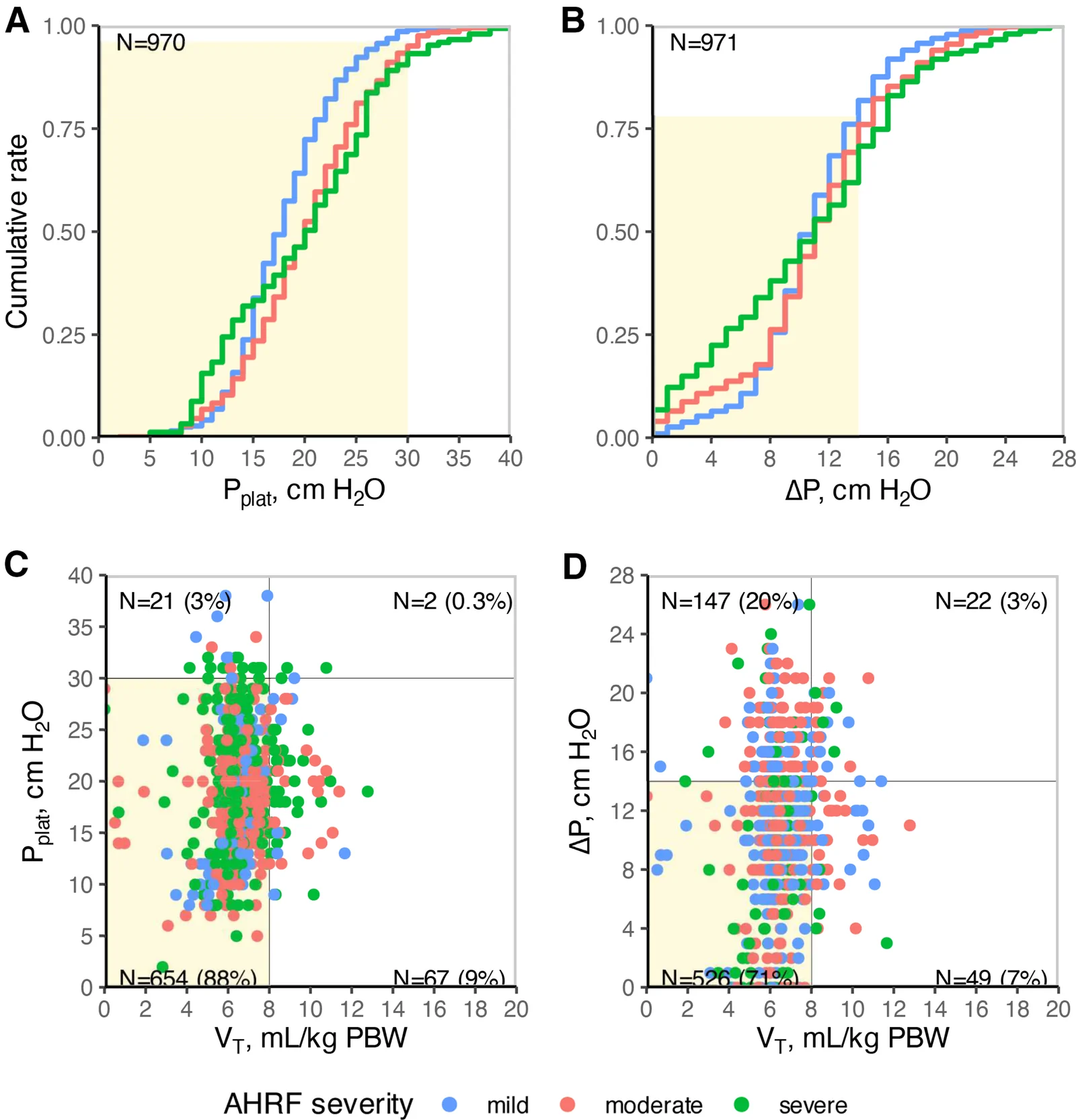
Baseline ventilatory parameters in patients receiving invasive mechanical ventilation with acute hypoxemic respiratory failure (AHRF), stratified by severity. *Cumulative frequency distribution plots* for plateau pressure (P_plat_; **A**) and driving pressure (∆P; **B**). A total of 933 patients (96%) had a P_plat_ less than or equal to 30 cm H_2_O, and 750 (77%) had a ∆P less than or equal to 14 cm H_2_O. Additionally, the distributions of tidal volume (Vt) vs. P_plat_ (**C**) and Vt vs. ∆P (**D**) are shown. Baseline measurements were collected at 8 am within the first 24 hr of invasive mechanical ventilation. AHRF severity was defined using Pao_2_/Fio_2_ thresholds. When unavailable, peripheral oxygen saturation/Fio_2_ thresholds were used. PBW = predicted body weight.

Among patients on IMV, vasopressors and chest tube placements were the most frequently used interventions, while neuromuscular blockade and extracorporeal life support (ECLS) were the most frequently used adjunctive therapies during the first 30 days of ICU stay (**eTable 3**, https://links.lww.com/CCM/I35). Use of renal replacement therapy, neuromuscular blockade, prone positioning, inhaled nitric oxide (iNO), and ECLS increased with greater AHRF severity. Among patients with severe AHRF, 26% received neuromuscular blockade, 13% received prone positioning, 12% received iNO, and 12% received ECLS within the first 30 days of ICU admission.

### ICU Length of Stay and Duration of IMV

The overall median (IQR) ICU LOS was 5 days (3–11 d), and 6 days (3–13 d) for patients on IMV (**Table 3** and **eTable 4**, https://links.lww.com/CCM/I35). Median (IQR) duration of IMV was 4 days (2–11 d).

**TABLE 3.** Outcomes of Patients With Acute Hypoxemic Respiratory Failure, Stratified by Severity

Outcomes	Overall (*n* = 10,832)	Stratification by Acute Hypoxemic Respiratory Failure Severity^a^			*p* ^b^
		Mild (*n* = 5,466)	Moderate (*n* = 4,070)	Severe (*n* = 1,296)	
ICU outcomes					
ICU length of stay, d	5 (3–11)	5 (2–9)	6 (3–13)	6 (2–15)	< 0.001
ICU mortality, *n*	2,539 (23%)	902 (17%)	1,072 (26%)	565 (44%)	< 0.001
30-d cumulative probability of events					
ICU discharge, %	74 (73–75)	82 (81–83)	70 (69–72)	52 (49–55)	< 0.001
Death, %	22 (22–23)	16 (15–17)	25 (24–26)	42 (39–44)	< 0.001

a Three thousand eight hundred thirty-one patients (35%) had missing Pao_2_/Fio_2_ at baseline and were classified using peripheral oxygen saturation (Spo_2_)/Fio_2_.

b Kruskal-Wallis test, Pearson χ^2^ test, and Gray test.

Values are reported as medians (interquartile range) or counts (%). Cumulative probabilities are shown as percentages (95% CI). Acute hypoxemic respiratory failure severity was defined using Pao_2_/Fio_2_ thresholds. When unavailable, Spo_2_/Fio_2_ thresholds were used.

### Mortality and VFD

Overall ICU mortality was 23% and increased with AHRF severity. Among 1054 patients (9.7%) receiving noninvasive respiratory support within 24 hours of ICU admission, the 30-day cumulative probability of death was 23% (95% CI, 20–25%; **Table 4**). Mortality increased and ICU discharge declined across worsening severity. Among 8280 patients (76%) receiving IMV within 24 hours of ICU admission, the 30-day cumulative probability of death was 25% (95% CI, 24–26%) and increased with severity (eTable 4, https://links.lww.com/CCM/I35). Patients with more severe AHRF, or with high baseline E_RS_, had fewer VFD, a lower probability of ICU discharge, and a higher probability of death at 30 days (**eTables** 4 and **5**, https://links.lww.com/CCM/I35).

**TABLE 4.** Outcomes of Patients on Noninvasive Respiratory Support With Acute Hypoxemic Respiratory Failure, Stratified by Severity

Outcomes	Overall (*n* = 1054)	Stratification by Acute Hypoxemic Respiratory Failure Severity^a^			*p* ^b^
		Mild (*n* = 381)	Moderate (*n* = 442)	Severe (*n* = 231)	
ICU outcomes					
ICU length of stay, d	5 (3–10)	4 (2–8)	5 (3–10)	7 (4–16)	< 0.001
ICU mortality, *n*	248 (24%)	55 (14%)	113 (26%)	80 (35%)	< 0.001
30-d cumulative probability of events					
ICU discharge, %	74 (71–77)	84 (80–87)	72 (67–76)	62 (56–68)	< 0.001
Death, %	23 (20–25)	14 (11–18)	25 (21–29)	32 (27–39)	< 0.001

a Four hundred eighty-eight patients (46%) had missing Pao_2_/Fio_2_ at baseline and were classified using peripheral oxygen saturation (Spo_2_)/Fio_2_.

b Kruskal-Wallis test, Pearson χ^2^ test, and Gray test.

Values are reported as medians (interquartile range) or counts (%). Cumulative probabilities are shown as percentages (95% CI). Acute hypoxemic respiratory failure severity was defined using Pao_2_/Fio_2_ thresholds. When unavailable, Spo_2_/Fio_2_ thresholds were used.

### Sensitivity and Subgroup Analyses

Restricting the cohort to patients with Spo_2_ less than 97% at baseline yielded a more hypoxemic population with less frequent IMV use (**eTable 6**, https://links.lww.com/CCM/I35). Outcomes were similar, with slightly higher mortality (**eTable 7**, https://links.lww.com/CCM/I35). Among IMV patients with plausible ARDS etiologies, baseline characteristics, ventilator settings, and outcomes were similar to the overall IMV cohort (**eTables 8–10**, https://links.lww.com/CCM/I35). In the subgroup of 1740 patients on IMV with Pao_2_/Fio_2_ less than 150 mm Hg, ARDS was documented in 282 patients (16%). Prone positioning was used in 183 patients (11%), including 104 (6%) with documented ARDS and increased over time, particularly after 2017 (**eFigs. 2** and **3**, https://links.lww.com/CCM/I35). Over the study period, respiratory support patterns were broadly similar, with a gradual increase in low-flow nasal cannula and HFNC use (**eFig. 4**, https://links.lww.com/CCM/I35). ICU LOS and IMV duration showed small variations. Oxygenation ratios were slightly lower after 2019. Vt and ∆P decreased modestly over time, with no major change in PEEP.

## DISCUSSION

In this large multicenter cohort of 21,714 adult ICU patients receiving supplemental oxygen or respiratory support for at least 4 hours, half met AHRF criteria within 24 hours of ICU admission. Overall ICU mortality was 23% and increased with AHRF severity. Among patients on IMV, ICU mortality increased with AHRF severity, while VFD and probability of ICU discharge declined. IMV duration and ICU LOS varied modestly by severity. Most patients on IMV received lung-protective ventilation. Use of adjunctive therapies increased with severity, although prone positioning remained infrequent despite greater utilization in later years. Outcomes among patients with Spo_2_ less than 97% were consistent with the full cohort, supporting our AHRF definition. Patients with plausible ARDS etiologies had similar baseline characteristics, ventilator settings, and outcomes compared with the overall IMV AHRF cohort.

Our findings align with prior studies reporting that around half of ICU patients present with AHRF early after admission (3, 6), and extend these observations to a large cohort spanning the full spectrum of respiratory support. Among patients initially managed with noninvasive respiratory support, oxygenation-based severity categories demonstrated a stepwise gradient in outcomes, supporting the clinical relevance of pragmatic Pao_2_/Fio_2_ or Spo_2_/Fio_2_-based stratification beyond invasively ventilated ARDS populations. Documented ARDS accounted for 8% of IMV patients with AHRF, compared with 20–25% in prior multinational cohorts (24). This likely reflects misclassification and incomplete ascertainment in routine practice, limited arterial sampling, and the increasing use of noninvasive respiratory support outside the Berlin framework (11, 25, 26). The newly proposed global ARDS definition now acknowledges the role of Spo_2_ when ABG analysis is not feasible, to help stratify disease severity (27), as well as to include nonintubated ARDS patients. Our findings further highlight that ABG measurements are frequently missing, which may further hinder ARDS recognition in routine practice. In our cohort, 35% of patients lacked Pao_2_/Fio_2_ data and were classified using Spo_2_/Fio_2_. Patients with AHRF resembled those reported in ARDS cohorts but were less hypoxemic (24). Mortality approached that reported in ARDS with progressive hypoxemia, reaching 47% in severe AHRF. This suggests an overlap between AHRF and ARDS while capturing additional high-risk patients not identified by the Berlin criteria. Among IMV patients with plausible ARDS etiologies, baseline characteristics, ventilator settings, and outcomes were comparable, suggesting etiology did not meaningfully influence management or prognosis. Our study supports the utility of a pragmatic definition of AHRF to identify patients who may benefit from ARDS-based interventions, even in the absence of formal ARDS diagnosis.

The Pao_2_/Fio_2_ ratio remains central to ARDS management as a prognostic marker and guide for adjunctive therapies (3, 16). However, ABG sampling is invasive and may yield variable results (13, 28, 29). In our cohort, over one-third of patients lacked Pao_2_/Fio_2_ measurements. Pulse oximetry offers a noninvasive, continuous, reliable, and cost-effective alternative for assessing oxygenation, demonstrating a strong quantitative correlation between Spo_2_/Fio_2_ and Pao_2_/Fio_2_ ratios (17, 30). Calibration bias may overestimate oxygenation in patients with darker skin tones, potentially misclassifying hypoxemia when Spo_2_/Fio_2_ is used as a surrogate (27). Recent ESICM guidelines acknowledge Spo_2_/Fio_2_ ratios as a surrogate for Pao_2_/Fio_2_ in assessing hypoxemia severity, especially when ABG is unavailable (8). Our findings support Spo_2_/Fio_2_ as a practical and accessible surrogate to identify patients with AHRF who might otherwise be overlooked, particularly in settings where ABG testing is unavailable or delayed.

Most IMV patients with available measurements were ventilated with lung-protective ventilation strategies developed for ARDS, and growing evidence suggests that these strategies also benefit the broader AHRF population (31, 32). Although limiting Vt and P_plat_ has been shown to reduce mortality in ARDS (33, 34), our findings suggest that these practices remain insufficiently integrated into routine care. Only 12% of patients on IMV had P_plat_ recorded within the first 24 hours, consistent with other large cohorts and highlighting persistent gaps in routine monitoring practices (24, 35). Even among patients ventilated in volume-controlled mode, P_plat_ was documented in only 38% of patients. Although the frequency of these measurements increased with AHRF severity, their overall underuse likely reflects missed opportunities to optimize lung-protective ventilation. This gap extends to E_RS_, which reflects the elastance of the respiratory system based on end-expiratory lung volume and determines the Vt delivered for a given ∆P, thereby reflecting the size of the aerated lung. High E_RS_ has been associated with increased mortality in ARDS (36, 37), and recent evidence suggests that Vt may need to be tailored to E_RS_, provided ∆P remains within protective limits (19). However, this interaction has not been evaluated in patients with AHRF. In our cohort, higher baseline E_RS_ was associated with worse outcomes, including prolonged IMV and increased mortality, supporting its value as a marker of severity. These findings mirror observations in ARDS and support the need to investigate personalized ventilation approaches based on lung mechanics, even outside traditional ARDS populations (38).

Our study has several strengths and limitations. Because iCORE includes ICU patients receiving supplemental oxygen or ventilatory support for at least 4 hours, estimates reflect AHRF burden within this respiratory support population rather than across all ICU admissions. iCORE is one of the largest multicenter Canadian databases on respiratory support, providing a detailed and comprehensive data source for investigating patients with AHRF. We used a pragmatic AHRF definition based on Pao_2_/Fio_2_ and Spo_2_/Fio_2_ thresholds that mitigated common diagnostic challenges associated with the ARDS definition (11, 25) and aligned more closely with how patients are stratified and managed in clinical practice. Oxygenation measurements were recorded once daily, so AHRF classification was based on a single value, which may have introduced severity misclassification. Sensitivity analyses showed consistent patterns in baseline characteristics and outcomes across strata, increasing confidence in the robustness of our definition. As with routinely collected data, missingness was present, particularly for ABG and static pressure measurements. However, this reflects real-world variability in monitoring practices and clinical decision-making. Future research should incorporate long-term follow-up to better define the impact of AHRF on prognosis.

## CONCLUSIONS

This large multicenter cohort study found that half of ICU patients receiving supplemental oxygen or respiratory support for at least 4 hours developed AHRF within the first 24 hours of ICU admission, with 76% requiring IMV. ICU LOS, duration of IMV, and mortality increased with AHRF severity. These findings support a pragmatic and operationally feasible definition of AHRF that reflects real-world clinical practice. Standardizing the definition of AHRF may improve patient identification and management, facilitate comparisons across studies, and optimize care.

## ACKNOWLEDGMENTS

We acknowledge all members of the Toronto Intensive Care Observational Registry project team for collecting and providing the data for this study.

## Supplementary Material
