# Supplementary material for: Epidemiology, Ventilatory Patterns, and Outcomes in Acute Hypoxemic Respiratory Failure Among ICU Patients Requiring Respiratory Support: A Registry-Based Cohort Study
Source: Crit Care Med. 2026 Aug 12;54(10):2601–12. doi: 10.1097/CCM.0000000000007284 (PMC13632807; doi:10.1097/CCM.0000000000007284)
Supplement: Supplementary file 1 [file ccm-54-2601-s001.pdf]

Epidemiology, ventilatory patterns, and outcomes in acute hypoxemic respiratory failure among ICU patients requiring respiratory support: a registry-based cohort study

ONLINE DATA SUPPLEMENT

Critical Care Medicine
2026

Stephan von Düring, MD MSc
Ewan Goligher, MD PhD
Andre Carlos Kajdacsy-Balla Amaral, MD
Gordon Rubenfeld, MD MSc
Niall D. Ferguson, MD MSc
Martin Urner, MD PhD
Eddy Fan, MD PhD

Correspondence to:
Eddy Fan, MD, PhD
Interdepartmental Division of Critical Care Medicine (IDCCM),
University of Toronto, Toronto, ON, Canada.

Table of contents

STROBE Statement Checklist of items that should be included in reports of cohort studies 3

eTable 1. Agreement between PaO_2/FiO_2 and SpO_2/FiO_2 severity classification among patients with AHRF and with paired measurements... 5

eTable 2. Baseline characteristics of patients on IMV with AHRF, stratified by severity 6

eTable 3. Interventions and adjunctive therapies performed during the first 30 days for patients on IMV with AHRF, stratified by severity.. 7

eTable 4. Outcomes of patients on IMV with AHRF, stratified by severity....................................... 8

eTable 5. Outcomes of patients on IMV with AHRF, stratified by respiratory system elastance normalized to the predicted body weight 9

eTable 6. Baseline characteristics of patients with AHRF and SpO_2 < 97%, stratified by severity. 10

eTable 7. Outcomes of patients with AHRF and SpO_2 < 97%, stratified by severity 11

eTable 8. Baseline characteristics of patients on IMV with plausible ARDS etiologies of AHRF, stratified by severity 12

eTable 9. Physiological and management characteristics of patients on IMV with plausible ARDS etiologies of AHRF, stratified by severity 13

eTable 10. Outcomes of patients on IMV with plausible ARDS etiologies of AHRF, stratified by severity.. 14

eFigure 1. Cumulative distribution of baseline V_T in patients receiving IMV for AHRF, stratified by severity.. 15

eFigure 2. Daily frequency of prone positioning during the first 30 days for patients on IMV with AHRF 16

eFigure 3. Temporal trends in prone positioning utilization among patients on IMV with AHRF 17

eFigure 4. Temporal trends in respiratory support, clinical course, gas exchange, and ventilation parameters in patients with AHRF 18

STROBE Statement Checklist of items that should be included in reports of ***cohort studies***

	Item No	Recommendation	Page No
**Title and abstract**	1	(*a*) Indicate the study's design with a commonly used term in the title or the abstract	1
		(*b*) Provide in the abstract an informative and balanced summary of what was done and what was found	3
**Introduction**			
Background/rationale	2	Explain the scientific background and rationale for the investigation being reported	5
Objectives	3	State specific objectives, including any prespecified hypotheses	6
**Methods**			
Study design	4	Present key elements of study design early in the paper	6
Setting	5	Describe the setting, locations, and relevant dates, including periods of recruitment, exposure, follow-up, and data collection	6
Participants	6	(*a*) Give the eligibility criteria, and the sources and methods of selection of participants. Describe methods of follow-up	6
		(*b*) For matched studies, give matching criteria and number of exposed and unexposed	-
Variables	7	Clearly define all outcomes, exposures, predictors, potential confounders, and effect modifiers. Give diagnostic criteria, if applicable	7
Data sources/ measurement	8*	For each variable of interest, give sources of data and details of methods of assessment (measurement). Describe comparability of assessment methods if there is more than one group	7
Bias	9	Describe any efforts to address potential sources of bias	8
Study size	10	Explain how the study size was arrived at	-
Quantitative variables	11	Explain how quantitative variables were handled in the analyses. If applicable, describe which groupings were chosen and why	-
Statistical methods	12	(*a*) Describe all statistical methods, including those used to control for confounding	8
		(*b*) Describe any methods used to examine subgroups and interactions	8
		(*c*) Explain how missing data were addressed	8
		(*d*) If applicable, explain how loss to follow-up was addressed	-
		(*e*) Describe any sensitivity analyses	8
**Results**			
Participants	13*	(a) Report numbers of individuals at each stage of study—eg numbers potentially eligible, examined for eligibility, confirmed eligible, included in the study, completing follow-up, and analysed	8
		(b) Give reasons for non-participation at each stage	8
		(c) Consider use of a flow diagram	8
Descriptive data	14*	(a) Give characteristics of study participants (eg demographic, clinical, social) and information on exposures and potential confounders	8
		(b) Indicate number of participants with missing data for each variable of interest	Supp
		(c) Summarise follow-up time (eg, average and total amount)	-
Outcome data	15*	Report numbers of outcome events or summary measures over time	9-10

Main results	16	(*a*) Give unadjusted estimates and, if applicable, confounder-adjusted estimates and their precision (eg, 95% confidence interval). Make clear which confounders were adjusted for and why they were included<br>(*b*) Report category boundaries when continuous variables were categorized<br>(*c*) If relevant, consider translating estimates of relative risk into absolute risk for a meaningful time period	-<br><br><br>8-10<br>-
Other analyses	17	Report other analyses done—eg analyses of subgroups and interactions, and sensitivity analyses	10
**Discussion**			
Key results	18	Summarise key results with reference to study objectives	10
Limitations	19	Discuss limitations of the study, taking into account sources of potential bias or imprecision. Discuss both direction and magnitude of any potential bias	12
Interpretation	20	Give a cautious overall interpretation of results considering objectives, limitations, multiplicity of analyses, results from similar studies, and other relevant evidence	10-12
Generalisability	21	Discuss the generalisability (external validity) of the study results	11-12
**Other information**			
Funding	22	Give the source of funding and the role of the funders for the present study and, if applicable, for the original study on which the present article is based	2

*Give information separately for exposed and unexposed groups.

Note: An Explanation and Elaboration article discusses each checklist item and gives methodological background and published examples of transparent reporting. The STROBE checklist is best used in conjunction with this article (freely available on the Web sites of PLoS Medicine at http://www.plosmedicine.org/, Annals of Internal Medicine at http://www.annals.org/, and Epidemiology at http://www.epidem.com/). Information on the STROBE Initiative is available at http://www.strobe-statement.org.

eTable 1. Agreement between PaO_2/FiO_2 and SpO_2/FiO_2 severity classification among patients with AHRF and with paired measurements

		PaO_2/FiO_2		
		**Mild**	**Moderate**	**Severe**
SpO_2/FiO_2	**Mild**	80.5	19.5	0
	**Moderate**	0	64.8	35.2
	**Severe**	0	0	100

Values are reported as row percentages.

N = 5,178 patients with paired PaO_2/FiO_2 and SpO_2/FiO_2 severity categories.
Overall agreement: 75.6%. Quadratic weighted kappa: 0.77

PaO_2, partial pressure of oxygen; FiO_2, fraction of inspired oxygen; SpO_2, peripheral oxygen saturation; AHRF, acute hypoxemic respiratory failure.

eTable 2. Baseline characteristics of patients on IMV with AHRF, stratified by severity

		Stratification by AHRF severity[a]		
	**Overall** (n = 8280)	**Mild** (n = 4065)	**Moderate** (n = 3247)	**Severe** (n = 968)
**Baseline characteristics**				
Age, years	62 (50, 72)	62 (50, 72)	62 (50, 72)	61 (48, 72)
Female, n	3,098 (37%)	1,528 (38%)	1,209 (37%)	361 (37%)
BMI, kg/m2	27 (23, 32)	27 (23, 32)	28 (23, 33)	28 (23, 33)
APACHE III score	78 (59, 97)	73 (54, 92)	80 (63, 99)	91 (70, 114)
SOFA score	6 (3, 9)	5 (3, 8)	7 (3, 9)	8 (4, 11)
**Baseline comorbidities**				
Cardiovascular disease, n	2,776 (34%)	1,416 (35%)	1,069 (33%)	291 (30%)
Respiratory disease, n	2,092 (25%)	1,006 (25%)	869 (27%)	217 (22%)
Diabetes, n	1,920 (23%)	924 (23%)	756 (23%)	240 (25%)
Active neoplasm, n	769 (9.3%)	354 (8.7%)	301 (9.3%)	114 (12%)
Chronic kidney disease, n	694 (8.4%)	344 (8.5%)	265 (8.2%)	85 (8.8%)
Liver failure, n	576 (7.0%)	297 (7.3%)	206 (6.3%)	73 (7.5%)
Immunosuppression, n	401 (4.8%)	169 (4.2%)	157 (4.8%)	75 (7.7%)
**Indication for respiratory support**				
Acute respiratory failure, n	5,897 (71%)	2,752 (68%)	2,397 (74%)	748 (77%)
Altered level of consciousness, n	2,526 (31%)	1,398 (34%)	899 (28%)	229 (24%)
Acute exacerbation of chronic pulmonary disease, n	207 (2.5%)	93 (2.3%)	98 (3.0%)	16 (1.7%)
Neuromuscular causes, n	45 (0.5%)	27 (0.7%)	12 (0.4%)	6 (0.6%)

AHRF severity was defined using PaO_2/FiO_2 thresholds. When unavailable, SpO_2/FiO_2 thresholds were used.

Values are reported as medians (IQR) or counts (%). Baseline data were collected at 8 AM within the first 24 hours of ICU admission.

IMV, invasive mechanical ventilation; AHRF, acute hypoxemic respiratory failure; BMI, body mass index; APACHE, acute physiology and chronic health evaluation; SOFA, sequential organ failure assessment; PaO_2, partial pressure of oxygen; FiO_2, fraction of inspired oxygen; SpO_2, peripheral oxygen saturation.

[a] 2455 (30%) patients had missing PaO_2/FiO_2 at baseline and were classified using SpO_2/FiO_2.

eTable 3. Interventions and adjunctive therapies performed during the first 30 days for patients on IMV with AHRF, stratified by severity

		Stratification by AHRF severity[a]			
	**Overall** (n = 8280)	**Mild** (n = 4065)	**Moderate** (n = 3247)	**Severe** (n = 968)	***p*-value[b]**
**Interventions**					
Vasopressors, n	5,163 (62%)	2,316 (57%)	2,089 (64%)	758 (78%)	<0.001
Steroids, n	193 (2.3%)	97 (2.4%)	70 (2.2%)	26 (2.7%)	0.6
Chest tube, n	1,106 (13%)	570 (14%)	414 (13%)	122 (13%)	0.2
Renal replacement therapy, n	665 (8.0%)	251 (6.2%)	292 (9.0%)	122 (13%)	<0.001
Tracheostomy, n	212 (2.6%)	108 (2.7%)	80 (2.5%)	24 (2.5%)	0.9
**Adjunctive therapies**					
Neuromuscular blockade, n	928 (11%)	219 (5.4%)	458 (14%)	251 (26%)	<0.001
Prone positioning, n	353 (4.3%)	46 (1.1%)	177 (5.5%)	130 (13%)	<0.001
iNO, n	434 (5.2%)	117 (2.9%)	200 (6.2%)	117 (12%)	<0.001
ECLS at admission, n	349 (4.2%)	118 (2.9%)	158 (4.9%)	73 (7.5%)	<0.001
ECLS at any time, n	522 (6.3%)	165 (4.1%)	237 (7.3%)	120 (12%)	<0.001

AHRF severity was defined using PaO_2/FiO_2 thresholds. When unavailable, SpO_2/FiO_2 thresholds were used.

All adjunctive therapies were recorded during the first 30 days of the ICU stay. Values are reported as medians (IQR) or counts (%).

IMV, invasive mechanical ventilation; AHRF, acute hypoxemic respiratory failure; iNO, inhaled nitric oxide; ECLS, extracorporeal life support; ICU, intensive care unit; PaO2, partial pressure of oxygen; FiO_2, fraction of inspired oxygen; SpO_2, peripheral oxygen saturation.

[a] 2,455 (30%) patients had missing PaO_2/FiO_2 at baseline and were classified using SpO_2/FiO_2.
[b] Fisher exact test

eTable 4. Outcomes of patients on IMV with AHRF, stratified by severity

		Stratification by AHRF severity[a]			
	**Overall** (n = 8280)	**Mild** (n = 4065)	**Moderate** (n = 3247)	**Severe** (n = 968)	***p*-value[b]**
**Outcomes**					
Duration of IMV, days	4 (2, 11)	4 (2, 9)	5 (2, 12)	5 (2, 13)	<0.001
Ventilator-free days at day 28, days	20 (0, 26)	23 (7, 26)	18 (-1, 25)	0 (-1, 21)	<0.001
ICU length of stay, days	6 (3, 13)	5 (3, 11)	7 (3, 14)	6 (2, 16)	<0.001
Duration of ECLS[c], days	8 (3, 16)	5 (2, 12)	8 (4, 18)	9 (4, 19)	0.01
ICU mortality, n	2,159 (26%)	785 (19%)	916 (28%)	458 (47%)	<0.001
**30-day cumulative probability of events**					
ICU discharge, %	71 (70, 72)	79 (77, 80)	68 (67, 70)	48 (45, 51)	<0.001
Receiving ECLS at any time[d], %	3.4 (2.8, 4.1)	1.5 (1.1, 2.1)	3.3 (2.6, 4.1)	5.9 (4.3, 8.0)	<0.001
Death, %	25 (24, 26)	19 (17, 20)	27 (25, 28)	45 (42, 48)	<0.001

AHRF severity was defined using PaO_2/FiO_2 thresholds. When unavailable, SpO_2/FiO_2 thresholds were used.

Values are reported as medians (IQR) or counts (%). Cumulative probabilities are shown as percentages (95% CI).

IMV, invasive mechanical ventilation; AHRF, acute hypoxemic respiratory failure; ICU, intensive care unit; ECLS, extracorporeal life support; PaO_2, partial pressure of oxygen; FiO_2, fraction of inspired oxygen.

[a] 2,455 (30%) patients had missing PaO_2/FiO_2 at baseline and were classified using SpO_2/FiO_2.
[b] Kruskal-Wallis test, Pearson Chi-squared test, and Gray test.
[c] Among 349 patients (4%) on ECLS at baseline, 118 (34%) were classified as mild, 158 (45%) as moderate, and 73 (21%) as severe AHRF.
[d] Among 522 patients (6%) that received ECLS at any time, 165 (32%) were classified as mild, 237 (45%) as moderate, and 120 (23%) as severe AHRF at baseline.

eTable 5. Outcomes of patients on IMV with AHRF, stratified by respiratory system elastance normalized to the predicted body weight

	Overall (n = 742)	Stratification by E_{RS}[a] Low (< 2.5) (n = 629)	High (≥ 2.5) (n = 113)	*p*-value[b]
**Outcomes**				
Duration of IMV, days	4 (2, 11)	4 (2, 11)	4 (2, 10)	>0.9
Ventilator-free days at day 28, days	19 (-1, 25)	20 (-1, 25)	15 (-1, 24)	0.014
ICU length of stay, days	5 (2, 12)	5 (2, 12)	5 (2, 11)	0.5
ICU mortality, n	213 (29%)	167 (27%)	46 (41%)	0.002
**30-day cumulative probability of events**				
ICU discharge, %	69 (65, 72)	70 (67, 74)	59 (49, 68)	0.036
Receiving ECLS at any time, %	6.8 (6.3, 7.3)	1.5 (0.80, 2.6)	0 (0, 0)	<0.001
Death, %	27 (24, 30)	25 (22, 29)	39 (30, 48)	0.003

Values are reported as medians (IQR) or counts (%). Cumulative probabilities are shown as percentages (95% CI).

IMV, invasive mechanical ventilation; E_{RS}, respiratory system elastance; ICU, intensive care unit; ECLS, extracorporeal life support.

[a] Among 8280 observations, 7538 (91%) had missing E_{RS} data.
[b] Kruskal-Wallis test, Pearson Chi-squared test, and Gray test.

eTable 6. Baseline characteristics of patients with AHRF and SpO_2 < 97%, stratified by severity

		Stratification by AHRF severity[a]		
	**Overall** (n = 5433)	**Mild** (n = 2020)	**Moderate** (n = 2432)	**Severe** (n = 981)
**Baseline characteristics**				
Age, years	63 (52, 73)	64 (54, 73)	63 (52, 73)	61 (50, 72)
Female, n	2,084 (38%)	775 (38%)	914 (38%)	395 (40%)
BMI, kg/m^2	28 (23, 33)	28 (24, 32)	28 (23, 33)	28 (23, 33)
APACHE III score	75 (58, 95)	70 (54, 87)	77 (60, 96)	85 (64, 110)
SOFA score	6 (3, 8)	4 (2, 6)	6 (3, 9)	7 (4, 11)
**Baseline comorbidities**				
Cardiovascular disease, n	2,092 (39%)	859 (43%)	916 (38%)	317 (32%)
Respiratory disease, n	1,515 (28%)	544 (27%)	731 (30%)	240 (24%)
Diabetes, n	1,368 (25%)	521 (26%)	614 (25%)	233 (24%)
Active neoplasm, n	605 (11%)	185 (9.2%)	276 (11%)	144 (15%)
Chronic kidney disease, n	488 (9.0%)	181 (9.0%)	219 (9.0%)	88 (9.0%)
Liver failure, n	381 (7.0%)	155 (7.7%)	160 (6.6%)	66 (6.7%)
Immunosuppression, n	316 (5.8%)	80 (4.0%)	134 (5.5%)	102 (10%)
**Indication for respiratory support**				
Acute respiratory failure, n	4,064 (75%)	1,450 (72%)	1,836 (75%)	778 (79%)
Altered level of consciousness, n	1,291 (24%)	543 (27%)	550 (23%)	198 (20%)
Acute exacerbation of chronic pulmonary disease, n	248 (4.6%)	85 (4.2%)	134 (5.5%)	29 (3.0%)
Neuromuscular causes, n	23 (0.4%)	12 (0.6%)	6 (0.2%)	5 (0.5%)
**Respiratory conditions**				
PaO_2/FiO_2, mm Hg	166 (115, 221)	243 (221, 273)	152 (128, 176)	78 (65, 90)
SpO_2/FiO_2	215 (153, 254)	268 (240, 313)	190 (160, 229)	100 (92, 120)
Non-invasive respiratory support, n	666 (12%)	180 (8.9%)	310 (13%)	176 (18%)
HFNC, n	224 (4.1%)	21 (1.0%)	101 (4.2%)	102 (10%)
CPAP, n	203 (3.7%)	99 (4.9%)	83 (3.4%)	21 (2.1%)
NIV, n	239 (4.4%)	60 (3.0%)	126 (5.2%)	53 (5.4%)
IMV, n	3,941 (73%)	1,363 (67%)	1,839 (76%)	739 (75%)

AHRF severity was defined using PaO_2/FiO_2 thresholds. When unavailable, SpO_2/FiO_2 thresholds were used.

Values are reported as medians (IQR) or counts (%). Baseline data were collected at 8 AM within the first 24 hours of ICU admission.

IMV, invasive mechanical ventilation; AHRF, acute hypoxemic respiratory failure; SpO_2, peripheral oxygen saturation; BMI, body mass index; APACHE, acute physiology and chronic health evaluation; SOFA, sequential organ failure assessment; PaO_2, partial pressure of oxygen; FiO_2, fraction of inspired oxygen; HFNC, high-flow nasal cannula; CPAP, continuous positive airway pressure; NIV, non-invasive ventilation.

[a] 1818 (33%) patients had missing PaO_2/FiO_2 at baseline and were classified using SpO_2/FiO_2.

eTable 7. Outcomes of patients with AHRF and SpO_2 < 97%, stratified by severity

	**Overall** (n = 5433)	Stratification by AHRF severity[a] **Mild** (n = 2020)	**Moderate** (n = 2432)	**Severe** (n = 981)	***p*-value[b]**
**Outcomes**					
ICU length of stay, days	5 (3, 12)	4 (2, 9)	6 (3, 13)	6 (2, 16)	<0.001
ICU mortality, n	1,432 (26%)	317 (16%)	648 (27%)	467 (48%)	<0.001
**30-day cumulative probability of events**					
ICU discharge, %	71 (69, 72)	83 (81, 84)	70 (68, 72)	48 (45, 51)	<0.001
Death, %	25 (24, 26)	15 (13, 17)	25 (24, 27)	45 (42, 49)	<0.001

AHRF severity was defined using PaO_2/FiO_2 thresholds. When unavailable, SpO_2/FiO_2 thresholds were used.

Values are reported as medians (IQR) or counts (%).

AHRF, acute hypoxemic respiratory failure; SpO_2, peripheral oxygen saturation; ICU, intensive care unit.

[a] 1818 (33%) patients had missing PaO_2/FiO_2 at baseline and were classified using SpO_2/FiO_2.
[b] Kruskal-Wallis test, Pearson Chi-squared test, and Gray test.

eTable 8. Baseline characteristics of patients on IMV with plausible ARDS etiologies of AHRF, stratified by severity

		Stratification by AHRF severity[a]		
	**Overall** (n = 4963)	**Mild** (n = 2348)	**Moderate** (n = 2004)	**Severe** (n = 611)
**Baseline characteristics**				
Age, years	61 (49, 71)	62 (50, 72)	61 (49, 71)	58 (48, 70)
Female, n	1,848 (37%)	887 (38%)	741 (37%)	220 (36%)
BMI, kg/m^2	27 (23, 32)	27 (23, 31)	28 (23, 33)	28 (23, 33)
APACHE III score	77 (60, 95)	73 (56, 91)	80 (63, 96)	85 (68, 105)
SOFA score	6 (3, 9)	5 (3, 8)	7 (4, 9)	7 (4, 11)
**Baseline comorbidities**				
Cardiovascular disease, n	1,575 (32%)	796 (34%)	619 (31%)	160 (26%)
Respiratory disease, n	1,351 (27%)	647 (28%)	563 (28%)	141 (23%)
Diabetes, n	1,088 (22%)	510 (22%)	433 (22%)	145 (24%)
Active neoplasm, n	466 (9.4%)	212 (9.0%)	184 (9.2%)	70 (11%)
Chronic kidney disease, n	375 (7.6%)	187 (8.0%)	144 (7.2%)	44 (7.2%)
Liver failure, n	342 (6.9%)	181 (7.7%)	118 (5.9%)	43 (7.0%)
Immunosuppression, n	262 (5.3%)	107 (4.6%)	100 (5.0%)	55 (9.0%)

Patients were excluded if they presented any of the following indications for respiratory support: neuromuscular causes, decreased level of consciousness, exacerbation of chronic pulmonary disease, exacerbation of chronic obstructive pulmonary disease, asthma, congestive heart failure, and malignancy.

AHRF severity was defined using PaO_2/FiO_2 thresholds. When unavailable, SpO_2/FiO_2 thresholds were used.

Values are reported as medians (IQR) or counts (%). Baseline data were collected at 8 AM within the first 24 hours of ICU admission.

IMV, invasive mechanical ventilation; ARDS, acute respiratory distress syndrome; AHRF, acute hypoxemic respiratory failure; BMI, body mass index; APACHE, acute physiology and chronic health evaluation; SOFA, sequential organ failure assessment; PaO_2, partial pressure of oxygen; FiO_2, fraction of inspired oxygen; SpO_2, peripheral oxygen saturation.

[a] 1352 (27%) patients had missing PaO_2/FiO_2 at baseline and were classified using SpO_2/FiO_2.

eTable 9. Physiological and management characteristics of patients on IMV with plausible ARDS etiologies of AHRF, stratified by severity

		Stratification by AHRF severity[a]		
	**Overall**	**Mild**	**Moderate**	**Severe**
	(n = 4963)	(n = 2348)	(n = 2004)	(n = 611)
**Blood gases**				
Arterial pH	7.35 (7.29, 7.40)	7.36 (7.31, 7.41)	7.34 (7.28, 7.40)	7.30 (7.21, 7.36)
$PaCO_2$, mm Hg	41 (36, 48)	40 (35, 45)	42 (36, 50)	47 (40, 57)
SpO_2 or SaO_2, %	96 (94, 98)	97 (96, 99)	96 (94, 98)	93 (91, 96)
PaO_2/FiO_2, mm Hg	194 (136, 245)	250 (225, 275)	155 (130, 180)	81 (69, 91)
SpO_2/FiO_2	200 (160, 245)	245 (237, 277)	190 (156, 202)	100 (93, 122)
**Ventilator parameters**				
FiO_2, %	50 (40, 60)	40 (35, 40)	50 (45, 60)	90 (75, 100)
Set respiratory rate, breaths/min	18 (14, 24)	16 (12, 22)	20 (16, 26)	24 (18, 30)
V_T, mL/kg PBW	6.5 (5.8, 7.7)	6.6 (5.9, 7.9)	6.5 (5.7, 7.6)	6.2 (5.3, 7.2)
Set PEEP, cm H_2O	8 (5, 10)	5 (5, 8)	10 (7, 12)	10 (8, 14)
PIP, cm H_2O	22 (17, 28)	20 (14, 24)	24 (19, 30)	29 (24, 33)
P_{plat}, cm H_2O	19 (15, 23)	18 (15, 22)	20 (16, 25)	19 (12, 26)
ΔP, cm H_2O	11 (8, 14)	11 (8, 14)	11 (8, 15)	10 (4, 15)
MP, J/min	20 (15, 28)	17 (13, 24)	22 (16, 29)	24 (19, 35)
Ventilatory ratio	1.58 (1.24, 2.02)	1.46 (1.15, 1.83)	1.65 (1.33, 2.11)	2.06 (1.49, 2.75)
E_{RS}, cm H_2O/(mL/kg PBW)	1.73 (1.23, 2.24)	1.72 (1.30, 2.17)	1.78 (1.22, 2.28)	1.59 (0.60, 2.22)
Pressure-controlled ventilation, n	1,936 (39%)	794 (34%)	844 (42%)	298 (49%)
Volume-controlled ventilation, n	1,397 (28%)	581 (25%)	599 (30%)	217 (36%)
Pressure-support ventilation, n	1,566 (32%)	953 (41%)	538 (27%)	75 (12%)
Other ventilation modes, n	43 (0.9%)	18 (0.8%)	17 (0.8%)	8 (1.3%)

Patients were excluded if they presented any of the following indications for respiratory support: neuromuscular causes, decreased level of consciousness, exacerbation of chronic pulmonary disease, exacerbation of chronic obstructive pulmonary disease, asthma, congestive heart failure, and malignancy.

AHRF severity was defined using PaO_2/FiO_2 thresholds. When unavailable, SpO_2/FiO_2 thresholds were used.

Values are reported as medians (IQR) or counts (%). Baseline data were collected at 8 AM within the first 24 hours of ICU admission.

IMV, invasive mechanical ventilation; ARDS, acute respiratory distress syndrome; AHRF, acute hypoxemic respiratory failure; $PaCO_2$, arterial partial pressure of carbon dioxide; SpO_2, peripheral oxygen saturation; SaO_2, arterial oxygen saturation; PaO_2, partial pressure of oxygen; FiO_2, fraction of inspired oxygen; V_T, tidal volume; PEEP, positive end-expiratory pressure; PIP, peak inspiratory pressure; P_{plat}, plateau pressure; ΔP, driving pressure; MP, mechanical power; E_{RS}, elastance of the respiratory system.

[a] 1352 (27%) patients had missing PaO_2/FiO_2 at baseline and were classified using SpO_2/FiO_2.

eTable 10. Outcomes of patients on IMV with plausible ARDS etiologies of AHRF, stratified by severity

	Overall	Stratification by AHRF severity[a]			*p*-value[b]
		**Mild**	**Moderate**	**Severe**	
	(n = 4963)	(n = 2348)	(n = 2004)	(n = 611)	
**Outcomes**					
Duration of IMV, days	5 (2, 11)	3 (2, 8)	6 (2, 13)	6 (2, 17)	<0.001
Ventilator-free days at day 28, days	21 (0, 26)	24 (14, 26)	19 (0, 25)	1 (-1, 22)	<0.001
ICU length of stay, days	6 (3, 14)	5 (3, 11)	7 (4, 16)	8 (3, 19)	<0.001
Duration of ECLS, days	8 (3, 18)	7 (2, 13)	9 (4, 18)	10 (4, 24)	0.04
ICU mortality, n	1,098 (22%)	350 (15%)	495 (25%)	253 (41%)	<0.001

Patients were excluded if they presented any of the following indications for respiratory support: neuromuscular causes, decreased level of consciousness, exacerbation of chronic pulmonary disease, exacerbation of chronic obstructive pulmonary disease, asthma, congestive heart failure, and malignancy.

AHRF severity was defined using PaO_2/FiO_2 thresholds. When unavailable, SpO_2/FiO_2 thresholds were used.

Values are reported as medians (IQR) or counts (%).

IMV, invasive mechanical ventilation; ARDS, acute respiratory distress syndrome; AHRF, acute hypoxemic respiratory failure; ICU, intensive care unit; ECLS, extracorporeal life support; PaO_2, partial pressure of oxygen; FiO_2, fraction of inspired oxygen; SpO_2, peripheral oxygen saturation.

[a] 1352 (27%) patients had missing PaO_2/FiO_2 at baseline and were classified using SpO_2/FiO_2.
[b] Kruskal-Wallis test, Pearson Chi-squared test, and Gray test.

eFigure 1. Cumulative distribution of baseline V_T in patients receiving IMV for AHRF, stratified by severity

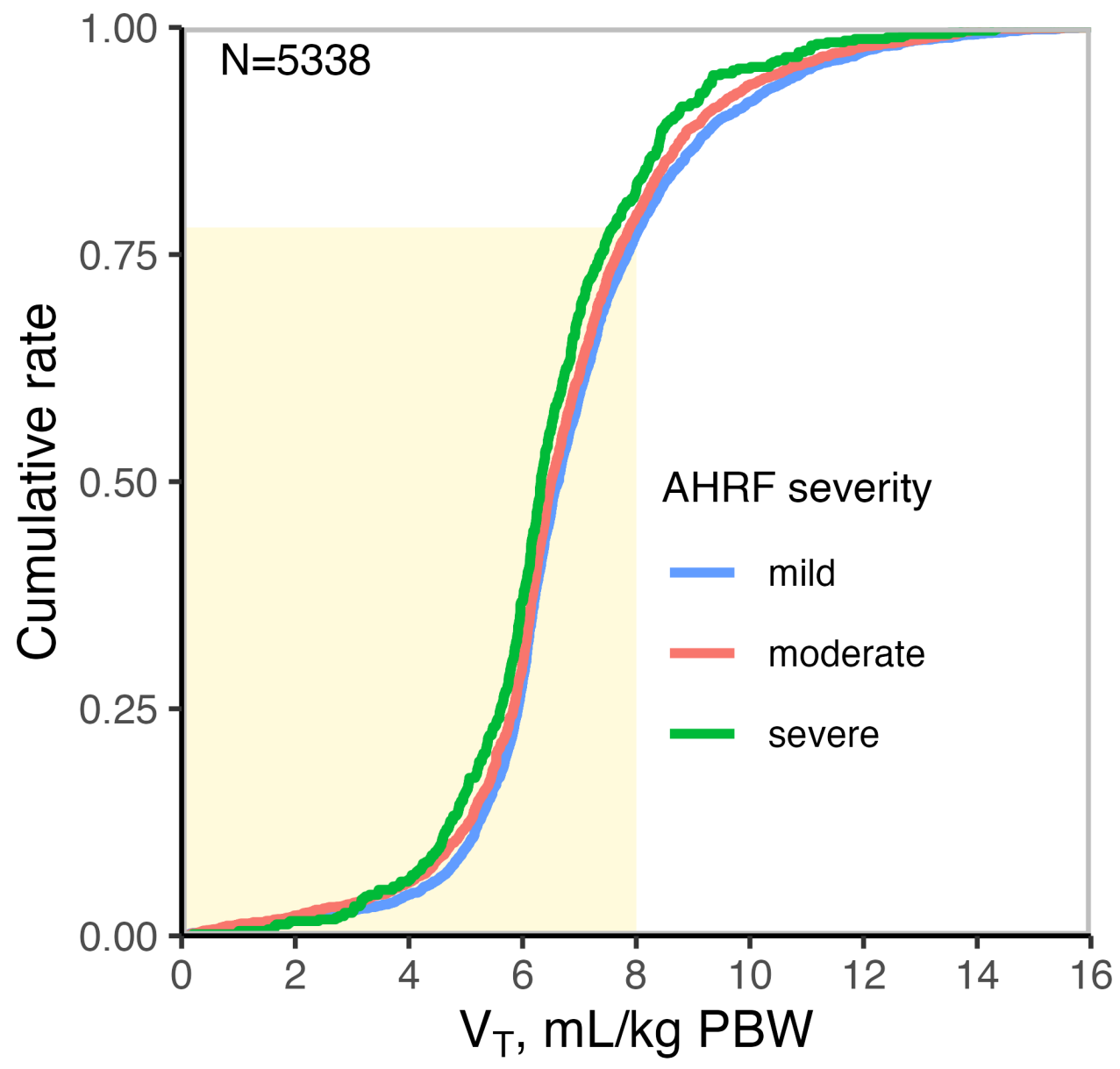

Overall, 4174 patients (78%) received $V_T \leq 8$ mL/kg PBW.

Baseline measurements were collected at 8 AM within the first 24 hours of IMV. AHRF severity was defined using PaO_2/FiO_2 thresholds. When unavailable, SpO_2/FiO_2 thresholds were used.

IMV, invasive mechanical ventilation; AHRF, acute hypoxemic respiratory failure; V_T, tidal volume; PBW, predicted body weight.

eFigure 2. Daily frequency of prone positioning during the first 30 days for patients on IMV with AHRF

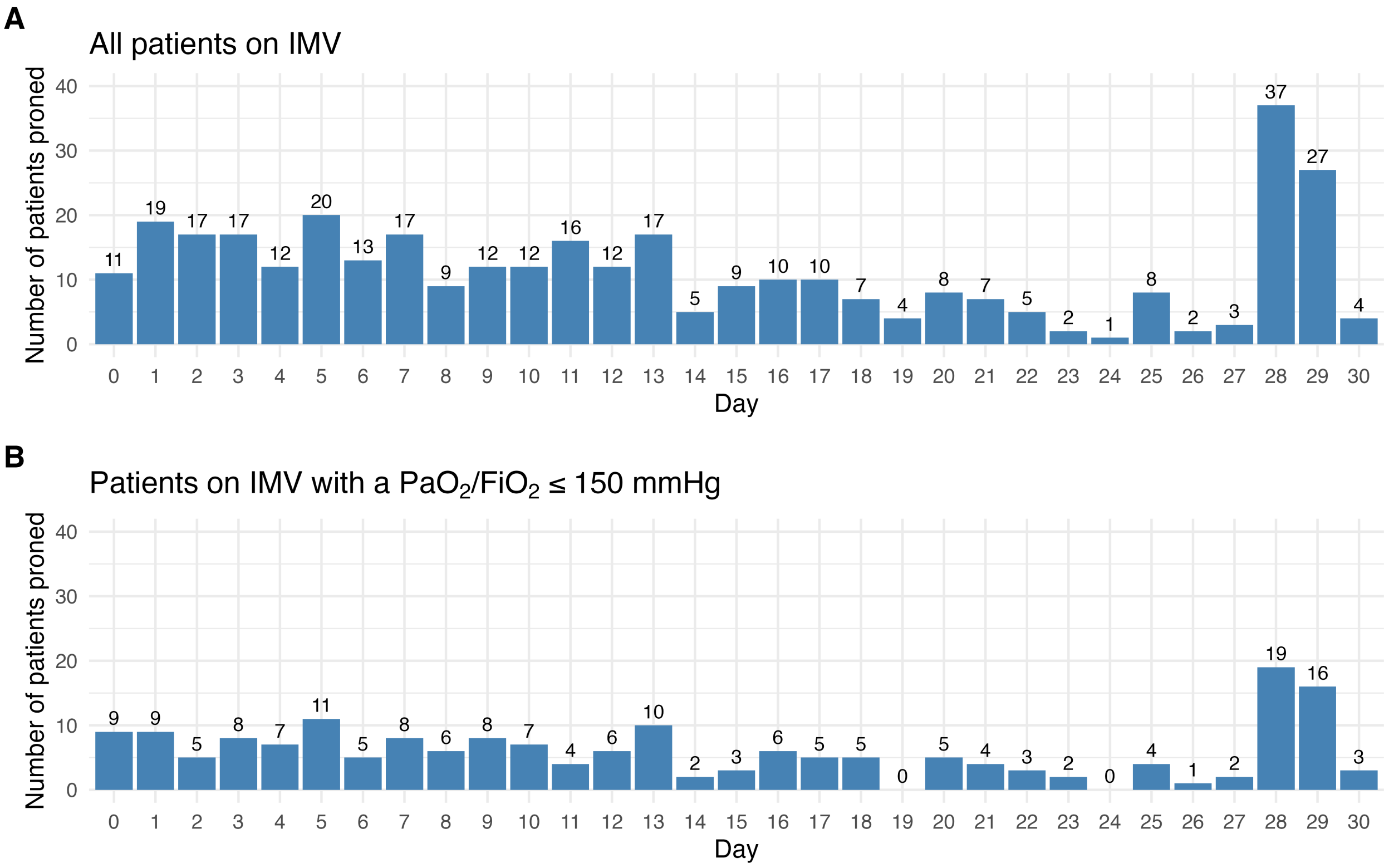

Number of patients who underwent prone positioning on each ICU day, from day 0 (ICU admission) to day 30. Panel **A** includes all 8280 patients on IMV, of whom 353 (4%) underwent prone positioning. Panel **B** shows the subset of 1740 patients with a $PaO_2/FiO_2 < 150$ mm Hg, of whom 183 (11%) underwent prone positioning. Most proning occurred within the first two weeks, with a secondary peak observed on days 28 and 29.

IMV, invasive mechanical ventilation; AHRF, acute hypoxemic respiratory failure; PaO_2, partial pressure of oxygen; FiO_2, fraction of inspired oxygen; ICU, intensive care unit.

eFigure 3. Temporal trends in prone positioning utilization among patients on IMV with AHRF

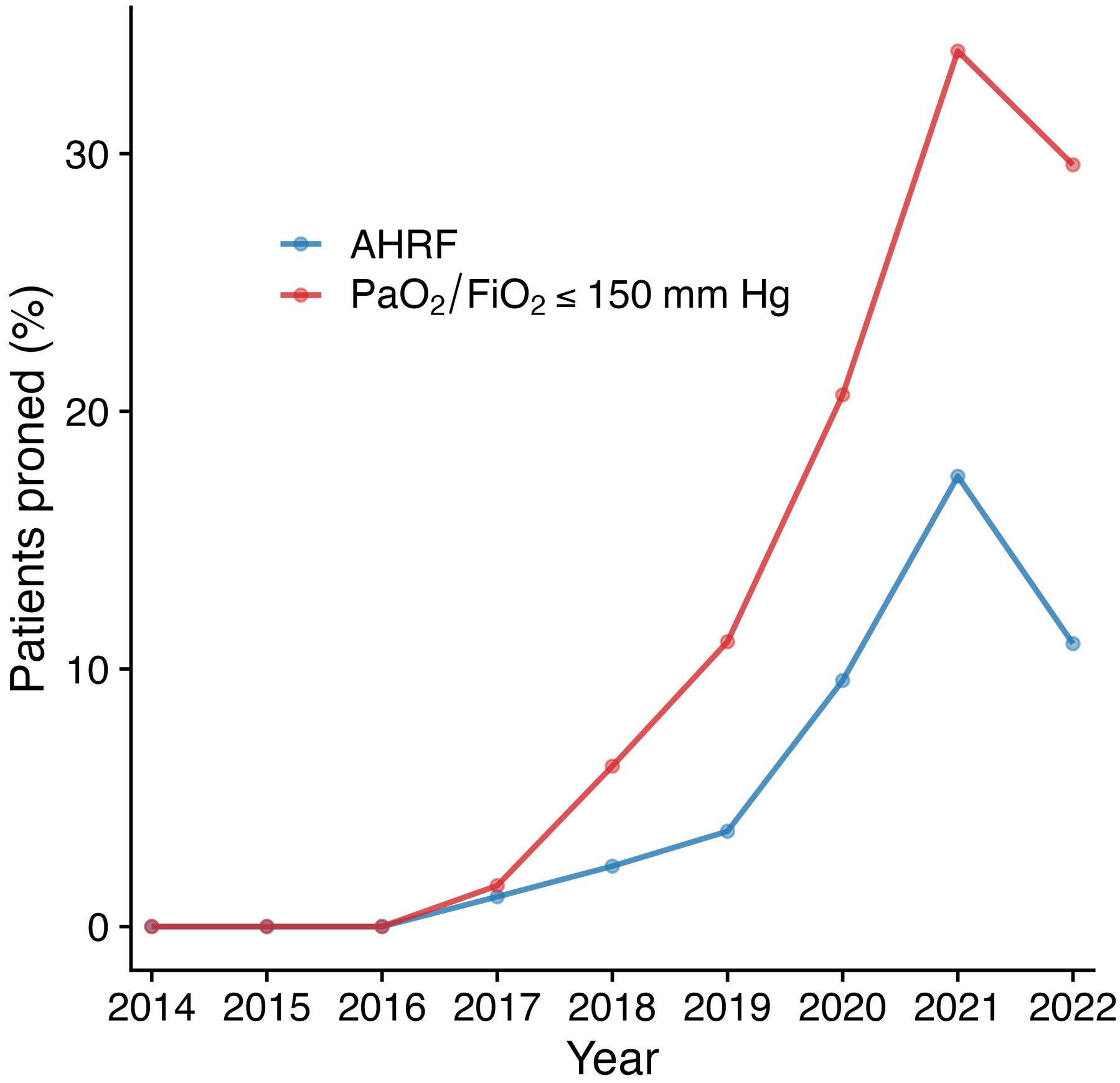

Yearly percentage of patients receiving IMV for AHRF who underwent prone positioning during their ICU stay, overall and among those with $PaO_2/FiO_2 \leq 150$ mm Hg.

IMV, invasive mechanical ventilation; AHRF, acute hypoxemic respiratory failure; PaO_2, partial pressure of oxygen; FiO_2, fraction of inspired oxygen; ICU, intensive care unit.

eFigure 4. Temporal trends in respiratory support, clinical course, gas exchange, and ventilation parameters in patients with AHRF

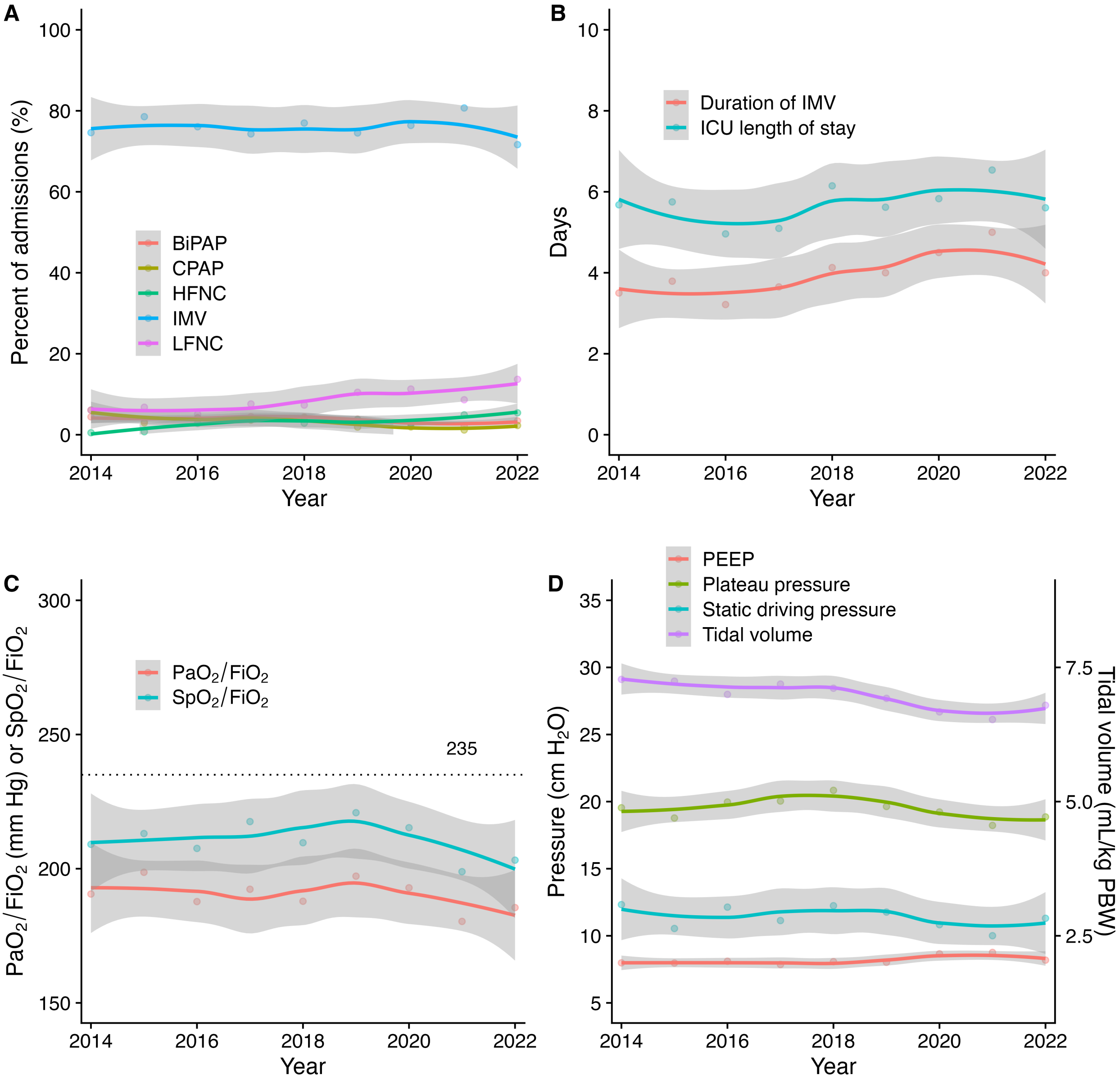

Temporal trends in respiratory management across the cohort. Panel **A** presents respiratory support strategies for all AHRF admissions. Panel **B** shows mean duration of IMV and ICU length of stay for all AHRF patients. Panel **C** displays mean PaO_2/FiO_2 and SpO_2/FiO_2 ratios in the AHRF cohort. Panel **D** reports mean ventilator settings, limited to patients receiving IMV. Baseline data were collected at 8 AM within the first 24 hours of IMV.

AHRF, acute hypoxemic respiratory failure; BiPAP, bilevel positive airway pressure; CPAP, continuous positive airway pressure; HFNC, high-flow nasal cannula; IMV, invasive mechanical ventilation; LFNC, low-flow nasal cannula; ICU, intensive care unit; PaO_2, partial pressure of oxygen; FiO_2, fraction of inspired oxygen; SpO_2, peripheral oxygen saturation; PEEP, positive end-expiratory pressure.
